# End-of-life care in a pediatric intensive care unit: the impact of the development of a palliative care unit

**DOI:** 10.1186/s12904-020-00575-4

**Published:** 2020-05-28

**Authors:** Sara Bobillo-Perez, Susana Segura, Monica Girona-Alarcon, Aida Felipe, Monica Balaguer, Lluisa Hernandez-Platero, Anna Sole-Ribalta, Carmina Guitart, Iolanda Jordan, Francisco Jose Cambra

**Affiliations:** 1 Disorders of Immunity and Respiration of the Pediatric Critical Patient Research Group, Institut Recerca Hospital Sant Joan de Déu, Universitat de Barcelona, Passeig Sant Joan de Déu, 2, Esplugues de Llobregat, 08950 Barcelona Spain; 2 Pediatric Intensive Care Unit Service, Hospital Sant Joan de Déu and University of Barcelona, Passeig Sant Joan de Déu, 2, 08950 Barcelona, Esplugues de Llobregat Spain; 3 Paediatric Infectious Diseases Research Group, Institut Recerca Hospital Sant Joan de Déu, CIBERESP, Passeig Sant Joan de Déu, 2, 08950 Esplugues de Llobregat, Barcelona Spain

**Keywords:** Hospital mortality, Palliative care, Pediatric intensive care units, Withdrawal, Withholding treatment

## Abstract

**Background:**

The purpose of this paper is to describe how end-of-life care is managed when life-support limitation is decided in a Pediatric Intensive Care Unit and to analyze the influence of the further development of the Palliative Care Unit.

**Methods:**

A 15-year retrospective study of children who died after life-support limitation was initiated in a pediatric intensive care unit. Patients were divided into two groups, pre- and post-palliative care unit development. Epidemiological and clinical data, the decision-making process, and the approach were analyzed. Data was obtained from patient medical records.

**Results:**

One hundred seventy-five patients were included. The main reason for admission was respiratory failure (86/175). A previous pathology was present in 152 patients (61/152 were neurological issues). The medical team and family participated together in the decision-making in 145 cases (82.8%). The family made the request in 10 cases (9 vs. 1, *p* = 0.019). Withdrawal was the main life-support limitation (113/175), followed by withholding life-sustaining treatments (37/175). Withdrawal was more frequent in the post-palliative group (57.4% vs. 74.3%, *p* = 0.031). In absolute numbers, respiratory support was the main type of support withdrawn.

**Conclusions:**

The main cause of life-support limitation was the unfavourable evolution of the underlying pathology. Families were involved in the decision-making process in a high percentage of the cases. The development of the Palliative Care Unit changed life-support limitation in our unit, with differences detected in the type of patient and in the strategy used. Increased confidence among intensivists when providing end-of-life care, and the availability of a Palliative Care Unit may contribute to improvements in the quality of end-of-life care.

## Background

Medical care standards in pediatrics are continuously improving. However, there are still situations in which a cure or an acceptable quality of life for our patients are not possible. Even with recent technological advances, sometimes we can only prolong the process of dying. It is necessary to weigh whether it is appropriate to maintain or begin an established treatment, or whether it would be more appropriate to remove it or not initiate it when this treatment is considered non-beneficial [1–3]. Each case should be evaluated individually, with the decision taken by consensus among all the professionals involved in the patient’s care and the family. The objective is to reach an agreement in which life support techniques/treatments are adapted to the situation of each patient [4]. The goals of care will thus change to ensure comfort rather than to provide a cure, and families must be made to understand that the best care for their child is being provided [5–7].

There is an increasing interest in end-of-life (EOL) care. In pediatrics, EOL care has progressively improved, especially with the development of palliative care that provides support at home or in the general ward. However, there are still EOL situations in which the intensivist must assess the futility of the established treatments and evaluate the appropriateness of a planned withholding or withdrawal of the life-support interventions if the patient’s situation so requires [5, 8, 9]. It should be noted that the clinician is not obliged to maintain a treatment that he or she considers non-beneficial, but must always seek consensus, to the best of their ability, with the family and other health professionals involved in the patient’s care. The family should be kept well informed, and the physicians must continue to provide the best possible comprehensive care and symptom management, for the family’s sake [3, 10, 11].

Both this study and our prior work [12] contribute to the wealth of published research regarding EOL in a Pediatric Intensive Care Unit (PICU), especially that regarding withdrawal of life-sustaining treatments. In 2011, a 7-year descriptive review was published by our institution [12]. The aim of that study was to describe the EOL decision-making process in a PICU. The present study aims to describe how EOL care in the PICU is carried out and how the implementation of palliative care has changed EOL practices in the PICU.

## Methods

**Fig. 1:**
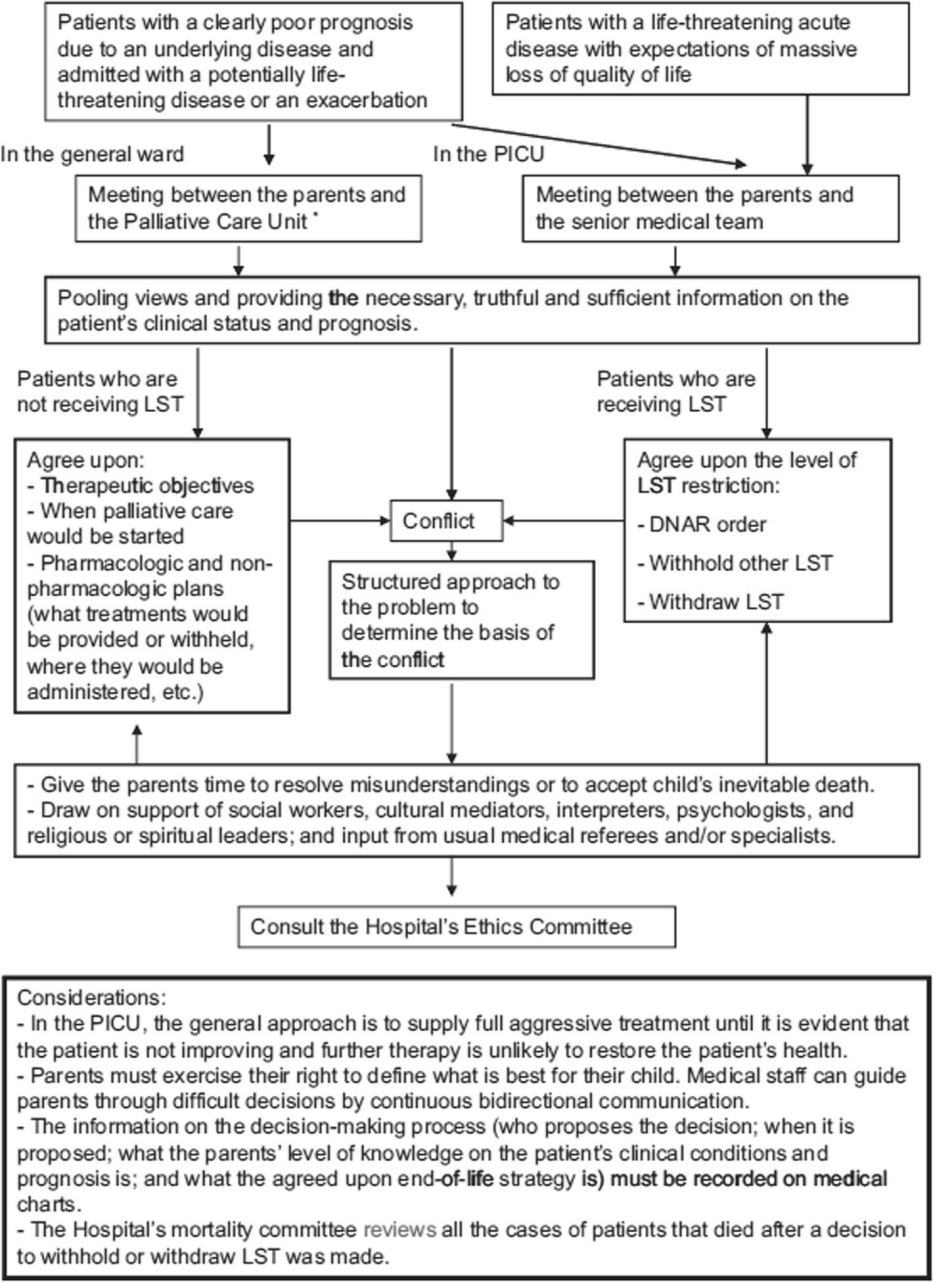
Algorithm for the end-of-life decision-making process at the unit. *The palliative care unit and the pediatric intensive care unit share the same physicians. PICU, Pediatric Intensive Care Unit; LST, life-sustaining treatment; DNAR, do not attempt resuscitation

This is a retrospective study performed in a referral tertiary pediatric hospital from 2001 to 2015. Patients who died in the PICU after the life-support limitation (LSL) decision were included. Patients who died after brain death or after unsuccessful cardiopulmonary resuscitation were excluded because LSL is not applicable. The unit’s specific algorithm for EOL care did not change over time (Fig. 1). During the study, there was no specific protocol for PICU admission criteria for these patients. The decision was individualized according to the situation of each patient.

Two groups were established: the pre-palliative group, which included patients who died between 2001 and 2008, and the post-palliative group, which included those who passed away between 2009 and 2015. The reason for this temporary division was the further development of the Palliative Care Unit in our hospital in 2009, when a specific multidisciplinary team was established consisting of doctors, nurses, and psychologists. Data from the pre-palliative group were partially published [12]. The hospital’s Ethics Committee and the Institutional Review Board approved the study. Informed consent was not collected because of the retrospective nature of the study.

LSL includes both the withdrawal or withholding of life-sustaining measures and do-not-resuscitate (DNR) orders. The withdrawal and withholding of life-sustaining measures have been well-defined in the literature since the 1990s [13]. Withdrawal of treatment was defined as discontinuing the life-sustaining intervention/treatment that was already in place. Withholding treatment was defined as the ‘non-initiation’ or the decision not to escalate a life-sustaining treatment [8, 14]. These two decisions were made after evaluating the patients’ prognosis and/or potential quality of life after surviving the critical episode. A poor prognosis was considered as the lack of an acceptable quality of life in the future after the critical episode. Each patient was evaluated individually by the medical team (including the nurse) together with the family. In this evaluation, the underlying disease, the prior quality of life, and the expected suffering secondary to the sequelae / pathology were considered.

Data collection was performed by reviewing the medical records. Epidemiological data and data about the decision-making process during EOL care were collected: gender, age, underlying disease, reason for admission, reason for LSL (the evolution of the underlying pathology, poor prognosis, neurological sequelae), who asked for LSL (medical team, family, both), need to consult the Ethics Committee, what was decided (withdrawal, withholding of support, or DNR order), how supports were withdrawn, and the sedation strategy. Due to our local protocol, all the information was recorded in detail by the physician responsible for the patient, who was the same physician who participated in the decision-making process and communicated with the family.

The categorical variables were expressed as frequency and percentage, and the continuous variables as median and interquartile range (IQR). The Chi-square test was used to compare categorical variables, and the Mann-Whitney U test was utilized for continuous variables. A value of *p* < 0.05 was considered statistically significant (SPSS23®).

## Results

**Fig. 2:**
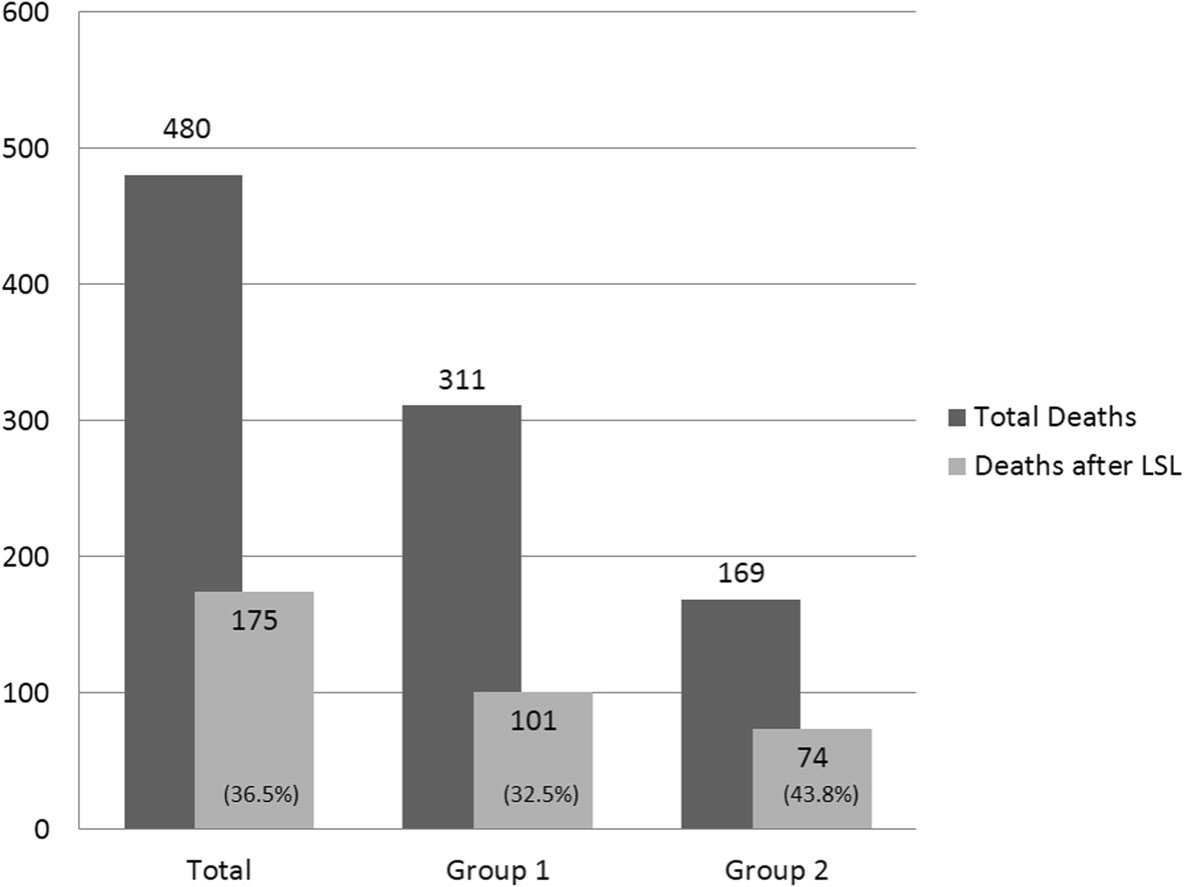
Distribution of patients in the different groups. The proportion of deaths after the life-support limitation (LSL) decision is included with respect to the total number of deaths (%)

During the 15 years of this study, 14,506 patients were admitted to the PICU and 480 patients died in the PICU (3.3%). LSL was decided in 175 patients, with this representing 36.5% of the deaths in the PICU. Figure 2 includes the distribution of overall deaths and deaths after LSL in the PICU. Eighty-nine (50.9%) of the children who died were female. The median age was 1 year old (IQR 0.3–4.65). A previous pathology was present in 152 patients (86.9%). The most frequent pathologies were neurological diseases (61 patients, 40.1%), followed by metabolic diseases (28 patients, 18.4%) and oncological diseases (22 patients, 14.5%). The main reason for admission was respiratory failure (86 patients, 49.1%). The median time spent in the PICU until death was 6 days (IQR 2–13). See Table 1.

**Table 1:** Summary of the main characteristics of the sample, including the two groups

General data	All (*n* = 175)	Pre-palliative group (*n* = 101)	Post-palliative group (*n* = 74)	p
Sex, male^a^	85 (48.6%)	46 (45.5%)	39 (52.7%)	0.420
Median age, years^b^	1 (0.3–4.65)	0.81 (0.31–5.43)	1 (0.38–4.25)	0.948
Underlying disease^a^	152 (86.9%)	88 (87.1%)	64 (86.5%)	0.901
Cardiac	22 (12.6%)	11 (10.9%)	11 (14.9%)	0.001
Respiratory	6 (3.4%)	0	6 (8.1%)	
Neurological	61 (34.9%)	43 (42.6%)	18 (24.3%)	
Oncologic	22 (12.6%)	11 (10.9%)	11 (14.9%)	
Metabolic	28 (16%)	20 (19.8%)	8 (10.8%)	
Others	13 (7.4%)	3 (3%)	10 13.5%)	
Reason for admission^a^				
Cardiac arrest	19 (10.9%)	16 (15.8%)	3 (4.1%)	0.328
Respiratory insufficiency	86 (49.1%)	45 (44.6%)	41 (55.4%)	
Cardiac decompensation	13 (7.4%)	7 (6.9%)	6 (8.1%)	
Sepsis	21 (12%)	13 (12.9%)	8 (10.8%)	
Intracranial hypertension	6 (3.4%)	3 (3%)	3 (4.1%)	
Others	29 (16.6%)	17 (16.8%)	12 (16.2%)	
Length of stay, days^b^	6 (2–13)	4 (2–12.5)	7 (3–14.25)	0.011
Reason for LSL^a^				
Unfavorable evolution of underlying disease	83 (47.4%)	51 (50.5%)	32 (43.2%)	0.319
Poor prognosis	54 (30.9%)	31 (30.7%)	23 (31.1%)	0.978
Neurological sequelae	35 (20%)	17 (16.8%)	18 (24.3%)	0.228
Life-support limitation^a^				
Withdrawal	113 (64.6%)	58 (57.4%)	55 (74.3%)	0.031
Withholding	37 (21.1%)	31 (30.7%)	6 (8%)	0.000
Do-not-resuscitate order	23 (13.1%)	10 (9.9%)	13 (17.6%)	0.152
Necropsy	64 (36.6%)	35 (34.6%)	29 (39.2%)	
Organ or tissue donation	13 (7.4%)	11 (10.9%)	2 (2.7%)	0.036

^a^Categorical variable, expressed as frequency (%), Chi-square test. ^b^Continuous variable, expressed as median (IQR), Mann-Whitney U test. LSL: life-support limitation

The most frequent reason for deciding LSL was the unfavorable evolution of the underlying pathology (83 patients, 47.4%). The main underlying pathology in these latter patients was neurological disease (38 patients, 46.3%), followed by metabolic disease (19 patients, 23.2%), oncological disease (7 patients, 8.5%), and cardiac disease (6 patients, 7.3%). The second most common reason for LSL was poor prognosis (54 patients, 30.9%) and the third was neurological sequelae (35 patients, 20%). There were no differences between the two groups as regards the reasons for deciding LSL, all with *p*-values of > 0.05 (data included in Table 1). The medical team proposed LSL in 145 cases (82.8%). The family made the request in 10 cases (5.7%), with differences between the groups: 9 patients (8.9%) in pre-palliative group versus 1 patient (1.4%) in post-palliative group (*p* = 0.019). There was no data about this in 18 cases (10.3%). The family agreed on the decision in the case of 157 patients (89.7%). The Ethics Committee was convened in 4 cases (2.3%), three of them in the pre-palliative group: in two cases the medical team did not reach a consensus with the family (a patient with vascular infiltration after the recurrence of an underlying neoplasia and a patient affected by Tay-Sachs disease with great neurological deterioration). The families requested the continuation of support / treatment that the medical team considered non-beneficial. In the other case, the request was made by the family to reaffirm the withdrawal action (limited to remove hydration and nutritional support). The last patient was under the guardianship of social services. Judicial intervention was not needed in any case.

Withdrawal was the most frequent LSL (113 patients, 64.6%). Statistically significant differences were detected between withdrawal and withholding over time (Table 1). There were no differences between previously healthy children and children with underlying diseases as regards the decision to withdrawal of support (in healthy children: 17, 77.3%; and in children with an underlying disease: 96, 63.6%, with *p* = 0.207) or DNR order (in healthy children: 4, 18.8%; and in children with an underlying disease: 19, 12.6%, with *p* = 0.470). However, there were differences between previously healthy children and children with underlying diseases as regards the decision to withhold support (in healthy children: 1, 4.5%; and in children with an underlying disease: 36, 23.8%, with *p* = 0.039).

Focusing on the withdrawal strategy (Table 2), the decrease in inotropic support was the main measure in terms of percentage (46 patients of the 71 who needed that support, 64.7%). Oxygen was required in 150 patients and mechanical ventilation in 128 and removed in 73 (48.7%) and 51 (39.8%), respectively.

**Table 2:** Life-sustaining treatments and devices required and removed

Support	All (*n* = 175)	Pre-palliative group groupgroupgroup (*n* = 101)	Post-palliative group (*n* = 74)	*p*
Inotropic				
Required	68 (38.9%)	45 (44.6%)	26 (35.1%)	
Removed	44 (64.7%)	32 (71.1%)	14 (53.8%)	0.046
Oxygen				
Required	142 (81.1%)	82 (81.2%)	68 (91.9%)	
Removed	71 (50.4%)	38 (46.3%)	35 (47.3%)	0.221
MV				
Required	121 (69.1%)	74 (73.3%)	54 (72.9%)	
Removed	49 (40.2%)	35 (47.3%)	16 (29.6%)	0.052
ECMO				
Required	3 (1.7%)	0 (0%)	3 (4.1%)	
Removed	3 (100%)	0	3 (100%)	0.039

Values expressed as frequency (percentage). *MV* mechanical ventilation, *ECMO* extracorporeal membrane oxygenation

In reference to treatment for analgesia and sedation, the combination of opioid drugs (morphine/fentanyl) and midazolam was the most frequent (92 patients, 52.6%), followed by opioids alone (19 patients, 10.9%), midazolam alone (12 patients, 6.9%), opioids and propofol (10 patients, 5.7%), midazolam with propofol (10 patients, 5.7%), propofol alone (6 patients, 3.4%), and none (4 patients, 2.3%). A necropsy was offered to all families and sixty-four accepted (36.6%). Thirteen patients became organ/tissue donors (7.4%).

## Discussion

This study provides the LSL experience in a single PICU and includes a large number of cases. A temporal division was used to split the sample into two groups. The reason for this division was to analyze the impact of improving palliative care in our center.

Worldwide, the number of deaths in PICUs decreased to less than 3% during the study window [15], similar to our data. A decrease in the number of deaths in the PICU over time was observed, with fewer deaths in the post-palliative group. However, the frequency of LSL with respect to the total number of deaths was higher in the post-palliative group. The training of the medical team in palliative care could explain this change. The LSL decision allows physicians to avoid providing non-beneficial treatments to patients at the end of their lives, and it is essential to identify which patients are in this situation. In 2009, the Palliative Care Unit of our center was improved. More pediatricians and nurses joined the unit and tele–health care and home visits were started. In some units, the EOL in the PICU is planned and guided by the palliative care team [16], although the recommendation nowadays is that intensivists be trained to assess and manage palliative care needs to provide a “good” EOL to the patients and their families [5, 17, 18]. In our unit, the intensivist managed EOL care. Palliative physicians and intensivists also work together in those situations in which intensive care could provide comfort at the end of a patient’s life. Some criteria for palliative care in the PICU were recently published [19]. Differences according to epidemiological characteristics between our results and other previous studies were not detected. The number of patients with underlying pathologies remained stable (85%) [20], however there were fewer patients with metabolic or neurological diseases in the post-palliative group, possibly due to the support of palliative care teams allowing patients to die in alternate locations, such as at home or in a general ward [21–23]. This improvement may reflect the work of the Palliative Care Unit, as they often facilitate EOL care at home or in the general wards.

The main reason for admission was respiratory failure, a weak point for patients with advanced chronic diseases [12, 24, 25]. The decision to initiate LSL was mainly conditioned by the evolution of the underlying disease, followed by a poor prognosis, without differences over time. As described in the recent report by Butt [26], the withdrawal of life-sustaining measures and the DNR order did not differ between previously healthy children and children with underlying diseases. However, withholding was more frequent in children with underlying diseases. The decision to withdraw treatments or to issue the DNR order is also difficult for families with previously ill children. By contrast, withholding can be planned beforehand in families with severely disabled children. LSL changed over time: the proportion of life-sustaining measures being withheld was higher in the pre-palliative group than in the post-palliative group, and this may be explained by the type of chronic patients in each group, with more patients having neurological and metabolic impairment in the pre-palliative group. The fact that the Palliative Care Unit was involved is important because non-admission to the PICU may also be seen as a measure of life support withholding. This change over time could also be justified by the training of the medical team in LSL. The withdrawal or withholding of life support are generally considered moral and ethical equals [27]. In recent years, the medical team has been inclined to attempt life-sustaining measures for a limited time. If a good response was not observed, then those life-sustaining measures were withdrawn. As in previous studies, families participated in the decision-making process for the LSL [24, 28]. A study published in 2004 that compared the decision-making process in northern and southern European countries suggested that there were differences in the information received by families because of the influence of the cultural roots of the different countries [25]. In our institution, the information was detailed and adapted to the degree of understanding of each family. Families participated in the LSL decision-making process in a high percentage of the cases. There are fewer requests for LSL from parents in recent years, possibly because the medical team has introduced LSL gradually over time. The flow of information also varied based on the capacity of the family to understand this information. There may be differences in opinion between the family and the clinicians regarding the optimal care for the child. The family may not accept the child’s situation and may request to continue with curative treatments instead of focusing on comfort and EOL care [29, 30]. The Ethics Committee was convened on only 4 occasions in the past 15 years, suggesting a high degree of agreement between families and the medical team. The Ethics Committee is a consultative and interdisciplinary group; it is formed by professionals trained in bioethics that advises on the resolution of ethical conflicts that may arise during healthcare provision. The decision of the Ethics Committee is not binding. Even so, in the two cases in which it was convened due to the lack of agreement between the medical team and the family, the Committee supported the decision made by the medical team and the families accepted that decision without the mediation of the courts or a judge’s order.

The time elapsed from PICU admission until the LSL decision varies according to the study reviewed. In some units, the median number of days is less than 3 [31] and in others it ranges to more than 3 weeks [24]. Cultural and religious issues may explain this variability. Our data showed less than 1 week until the LSL. The most frequent mode of death was via the withdrawal of life support, similar to the data reported by Zawitowski [24]. This differs from other studies [26, 32, 33], perhaps because of cultural differences and the evolution of LSL practices in the PICU. In absolute numbers, the support most commonly removed was respiratory support, similar to other publications [34]. Inotropic support was withdrawn in up to 65% of patients requiring it. These data are similar to those reported in previous studies [24]. Extracorporeal life support was withdrawn in all cases. As has been observed in other studies, the withdrawal of medically administered nutrition support was not generally considered in the PICU [35–37]. Sedation to ensure a dignified EOL for these patients is a fundamental concern. As in other studies [12, 24], opioids were the main analgesics used. They were used in conjunction with midazolam in more than 50% of the cases. Four patients received no sedatives, all of them with severe neurological damage. No patients were treated with neuromuscular blocking agents during the EOL process, which is a controversial treatment [20, 38]. No specific sedation scale for EOL was used in this study.

The main limitation of this study is its retrospective design. This factor could make the results quite variable, as data may not be accurately reflected in the medical documentation. Also, the granularity of the data and the single-center design are limitations, although this confers homogeneity to the study: the same standardized approach for determining the goal of EOL care was applied. It is important to note that the analysis of the impact of the Palliative Care Unit in a hospital is not complete without taking into account those patients who are managed in a general hospital ward or at home. However, this study focuses on patients cared for in the PICU, since the development of the Palliative Care Unit also influences care within this setting.

In conclusion, fewer children died in our PICU in recent years, and the LSL proportionally increased, although the percentage is still relatively low. A large percentage of the cases had a chronic disease. The main cause of LSL in the PICU was the unfavorable evolution of the underlying pathology. Families were involved in the EOL decision-making process. Withdrawal was the most frequent LSL. Withdrawing mechanical ventilation and oxygen were the main actions taken. Undoubtedly, the focus on holistic, compassionate, and child-centered care within the PICU helps achieve a dignified EOL in which the parents are also involved in the decision-making process and the care provided to their children. Increased confidence among the clinicians working in the PICU when providing EOL care, as well as the availability of a Palliative Care Unit, contribute to this improvement in quality EOL care.

## Data Availability

The datasets used and/or analysed during the current study are available from the corresponding author on reasonable request.

## Abbreviations

DNR: Do-not-resuscitate
EOL: End-of-life
LSL: Life-support limitation
PICU: Pediatric Intensive Care Unit
